# Finite Element Modeling of CNS White Matter Kinematics: Use of a 3D RVE to Determine Material Properties

**DOI:** 10.3389/fbioe.2013.00019

**Published:** 2013-12-09

**Authors:** Yi Pan, Daniel Sullivan, David I. Shreiber, Assimina A. Pelegri

**Affiliations:** ^1^Department of Mechanical and Aerospace Engineering, Rutgers, The State University of New Jersey, Piscataway, NJ, USA; ^2^Department of Biomedical Engineering, Rutgers, The State University of New Jersey, Piscataway, NJ, USA

**Keywords:** multi-scale modeling, microstructural properties, traumatic brain injury, spinal cord injury, axonal injury

## Abstract

Axonal injury represents a critical target area for the prevention and treatment of traumatic brain and spinal cord injuries. Finite element (FE) models of the head and/or brain are often used to predict brain injury caused by external mechanical loadings, such as explosive waves and direct impact. The accuracy of these numerical models depends on correctly determining the material properties and on the precise depiction of the tissues’ microstructure (microscopic level). Moreover, since the axonal microstructure for specific regions of the brain white matter is locally oriented, the stress, and strain fields are highly anisotropic and axon orientation dependent. Additionally, mechanical strain has been identified as the proximal cause of axonal injury, which further demonstrates the importance of this multi-scale relationship. In this study, our previously developed FE and kinematic axonal models are coupled and applied to a pseudo 3-dimensional representative volume element of central nervous system white matter to investigate the multi-scale mechanical behavior. An inverse FE procedure was developed to identify material parameters of spinal cord white matter by combining the results of uniaxial testing with FE modeling. A satisfactory balance between simulation and experiment was achieved via optimization by minimizing the squared error between the simulated and experimental force-stretch curve. The combination of experimental testing and FE analysis provides a useful analysis tool for soft biological tissues in general, and specifically enables evaluations of the axonal response to tissue-level loading and subsequent predictions of axonal damage.

## Introduction

An increase in the incidence and improvements in the diagnosis of traumatic brain injury (TBI) have increased awareness of the public about TBI’s serious health effects. Axonal injury, which is considered to be a major contributor to cognitive dysfunction following TBI, represents a critical focal area for TBI and spinal cord injury (SCI) prevention and treatment. FE models (FEM) of the head and/or brain are often used to predict brain injury caused by mechanical loading. Many attempts have been made to understand the injury mechanism and to define the mechanical parameters that govern axonal injury. Mechanical strain has been identified as the proximal cause of axonal injury (Wright and Ramesh, 2012), while secondary ischemic and excitotoxic insults associated with the primary trauma potentially exacerbate the structural and functional damage. Efforts to simulate the deformation, stress, and strain fields of brain tissues using computational models have primarily treated brain tissues as homogeneous materials, albeit with potential differences in properties between gray and white matter. As such, stress and strain fields were obtained in an average sense, which can be interpreted as macroscopic behavior (Kleiven, 2006; Ho and Kleiven, 2007; Cloots et al., 2008; Mao et al., 2010).

There have been many reports on the mechanical characterization of central nervous system (CNS) tissue. For example, Miller et al. (2000) performed an *in vivo* indentation experiment to obtain a force-displacement curve, which was reproduced by using a three dimensional, non-linear FEM. By appropriately increasing material parameters describing the instantaneous stiffness of the tissue, they were able to reproduce the *in vivo* experimental curve. Shreiber et al. (2009) performed experiments to demonstrate the influence of myelin and glia on the tensile properties of the embryonic day 18 (E18) chick embryo spinal cord. They found that myelin and cellular coupling of axons via the glial matrix dictates the tensile response of the tissue. This work is an example of the influence of microstructural kinematics of CNS white matter that was introduced by Bain et al. (2003). The effects of non-linear material properties and the convoluted structure of the vasculature was investigated by Ho and Kleiven (2007) by using FEM and comparing the results with the 3D FEMs. Compared to a model without vasculature, the peak average strain reduction in a model with non-linear and linear elastic vasculature was 2 and 5%, respectively, indicating that the influence of the vasculature on the dynamic response of the brain is minimal.

Finite element (FE) analysis has become an important numerical tool for studying not only the mechanical behavior of brain tissues, but also prediction of TBI. Brain tissue has typically been characterized as a hyperelastic material (Meaney, 2003; Shreiber et al., 2009) and viscoelastic material (Horgan and Gilchrist, 2003; Post et al., 2012). For example, Post et al. (2012) used a linearly viscoelastic material model combined with a large deformation theory to model brain tissue within a model of the head. The strain and Von Mises stress within the brain were extracted after simulating impacts that emulated typical loading curve shapes and peaks. However, the relationships between these bulk metric of the stress and strain fields within the brain relate to specific TBI neuropathologies and functional outcomes, such as concussion. Some efforts have been devoted toward this end. Sayed et al. presented a biomechanical model for TBI and soft tissue damage. A variational constitutive model for soft biological tissues was utilized to reproduce axonal damage and cavitation injury through inelastic deformation (Sayed et al., 2008). Mechanical damage of brain tissue was classified as volumetric (compression/tension) and shear-type which were associated with intracranial pressure and shear stresses, respectively.

The aforementioned FEM work is based on homogenized assumptions of brain tissue properties. However, the orientation and kinematic behavior of the microstructural constituents of white matter – including axons and the surrounding glia and vasculature – introduce significant anisotropy and complex bulk behavior. Thus, the accuracy, and perhaps ultimately the utility of FEM simulations depends not only on correctly determining the material properties, but also on the precise depiction of the tissues’ microstructure (microscopic level including axon, glial cell, and blood vessels, etc.), to effectively relate the bulk tissue response to the actual elements that are injured. Recent computational studies have recognized the potential importance of this multi-scale behavior. Karami et al. (2009) performed FE analysis (FEA) using a unit-cell comprised of undulated axons embedded within and fully coupled to a glial matrix. Their micromechanical FEM provides insight into how the local stress and strain fields are affected by the tortuosity (which we use interchangeably with undulation) of an axon, which is defined as the total axon path length divided by its end-to-end length. However, experimental characterizations by Bain et al. (2003) and Hao and Shreiber (2007) clearly demonstrate that axons are not fully coupled to the glial matrix, and instead demonstrate complex, strain-dependent coupling behavior. Pan et al. (2011) developed a unit-cell model that included multiple axon segments with different tortuosities and coupling that emulated experimental data (Bain et al., 2003). The extended unit-cell model captured the kinematic behavior of axons very well when compared with experimental results. They found that the degree of coupling within the tissue affects the continuum mechanical properties of the tissue. Cloots et al. (2011) developed another micromechanical model to study how axonal strain is affected by the inclusion of other local cells. Various factors, such as the inclusion of stiffness with respect to the surrounding tissue, the axonal orientation, and the maximum diversion angle of the axons, significantly influenced the axonal strain.

Although the extended unit-cell model by Pan et al. (2011) successfully captured the kinematics behavior of axons, the model was simplified and did not effectively allow representation of the wide distribution of axon tortuosity that is evident in white matter. To overcome this limitation, a pseudo-3D model is proposed in this study. A pseudo-3D representative volume element (RVE) is built using a random process. Its axons, resembling the axonal structure of CNS white matter, are embedded into an extracellular matrix (ECM). The kinematic axonal coupling model, which demonstrated good correlation to experimental observations in our previous study, is applied to the RVEs in the FEM, and the tissue-level mechanical response is investigated. The FE model employs our previously published approach to introduce strain-dependent kinematic coupling of axons to matrix and published experimental results for mechanical properties.

## Materials and Methods

An inverse FE procedure is used to identify the material parameters of spinal cord white matter by combining uniaxial testing and FEM. An objective function is selected for minimization through which material parameters are determined. Hereafter, the methods employed to this end are presented.

### Representative volume element of CNS white matter

#### RVE construction

**Figure 1:**
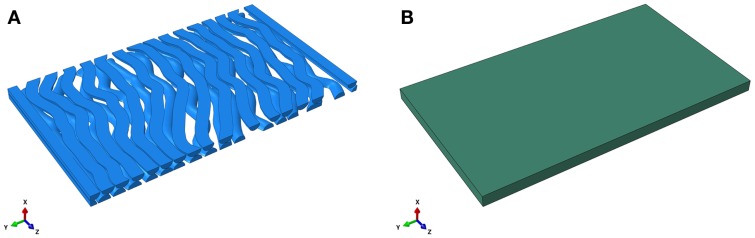
**Finite element model of the undulated axons (A) and the extracellular matrix (B)**.

**Figure 2:**
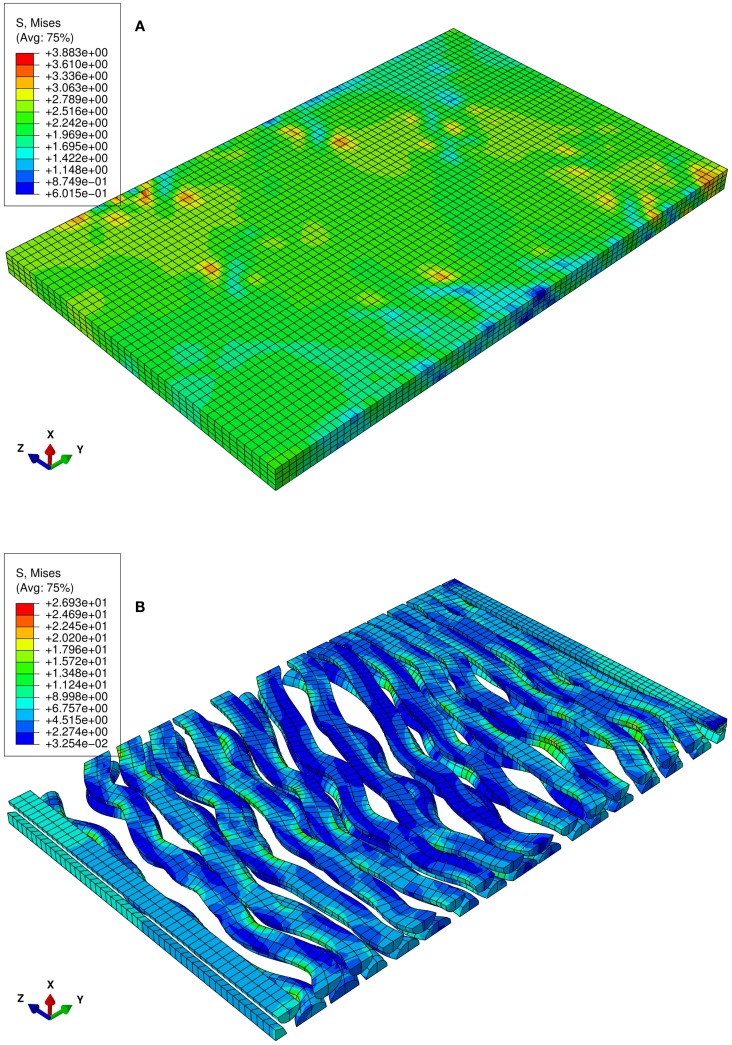
**Numerical uniaxial tensile experiment at the stretch level of 1.06**. **(A)** von Mises stress contour of the matrix of the RVE in which the undulated axons are embedded. **(B)** von Mises stress contour of the axons. Highly localized stress implies the effect of the axon geometry on local stress and strain field.

The FE model of an RVE is developed using ABAQUS 6.11 and Python scripting. An RVE is a sub-domain of a composite material whose size needs to be sufficiently large to include necessary fiber information such that the macroscopic homogeneous mechanical behavior of the composite material can be derived. To reduce computational demands in a FEA, one might need to limit the size of an RVE while retain necessary fibers. The interested reader is referred to Iorga et al. (2008). In this study, a pseudo-3D RVE for the CNS white matter is composed of many undulated axons that are embedded in the ECM. A cuboid (*x* = 0.4 μm, *y* = 10 μm, *z* = 5.68 μm) is created representing the matrix surrounding the axons as shown in Figure 1. The axons are undulated and their geometry is complex. The undulation varies from axon-to-axon as well as along an individual axon. The distribution of undulation of the axons within the RVE model is based on those found by Bain et al. (2003). An undulated axon is represented by a poly-line and a cubic spline: a poly-line is composed of multiple connecting line segments extending from random points on one face of the cuboid (*z* = 0 plane) to its opposite face (*z* = 5.68 plane); and the cubic spline, representing the backbone of an axon, is generated by depicting every key point on the poly-line. Sweeping a circle along the cubic spline then generates the axons. In this study, the diameter of an axon is fixed to 0.4 μm, although it may vary from axon-to-axon in the range of sub-micron to about 20 μm for porcine optic and sciatic nerves (Assaf et al., 2008). The portion of an axon that falls outside of the cuboid is trimmed. The volume fraction of axons is set to 53% (Karami et al., 2009). The total number of axons in the matrix is 33, which is determined by the volume fraction, as shown in Figure 2. The average undulation of the axons ranges from 1.05 to 1.25.

#### Kinematic of CNS white matter

In this pseudo-3D RVE model, the axons interact with the surrounding matrix via the embedded element approach in ABAQUS. Embedded elements allow the introduction of an axon fiber within a matrix without removing the matrix material where the fiber resides. If the elements representing the fiber are not specified as embedded, there is no interaction between the matrix and fiber. In our model, this coupling is governed by the axon transitional kinematic model (TKM) (Pan et al., 2011), a model of axon-ECM interaction derived from statistical analysis of experimental data (Bain et al., 2003). Specifically, a single axon was divided into 50 sub-segments along the axial direction. According to the TKM, the percentage of an axon that is embedded in the matrix depends on its current undulation and applied stretch (Pan et al., 2011). In general, there is a transition undulation that dictates whether an axon is coupled or not. As stretch is applied, the axons straighten and undulation decreases. Accordingly, the likelihood of reaching the transition undulation, thereby coupling the deformation of the axon to the matrix, increases with increasing stretch. If the undulation of an axon is less than 1.08, the percentages of that axon being coupled are 8, 20, and 44% of its whole length when the corresponding applied stretches are less than 1.06, 1.12, and 1.25, respectively. If undulation is greater than 1.08, the axon does not interact with the matrix (except both ends are tied to the matrix at the RVE surfaces). The threshold undulation, 1.08, was determined from experimental data by Bain et al. (2003).

To demonstrate our model, three RVEs (RVE1, RVE2, RVE3) with randomly generated axons were simulated. Initially, the models were loaded with applied stretches ranging from 1.0 to 1.06 in the *z*-direction, which was done by fixing *U_z_* = 0 on the *Z* = 0 face and applying the displacement, *U_z_*_a_, on the opposite face. Next, this was done with a range of 1.06–1.12 and finally with a range of 1.12–1.25. The tortuosity of each axon at the initial stage (λ = 1.0) and after each step (λ = 1.06, 1.12, and 1.25) was computed to compare to the experimental data and previous modeling approaches. Interactions with embedded elements were updated after each step.

### Inverse FE procedure

An inverse FE procedure was developed to identify material parameters of spinal cord white matter by combining uniaxial testing and FEA using the pseudo-3D RVE. The material properties of axons and ECMs were modeled with Ogden’s isotropic, large deformation, hyperelastic material model in the strain energy form (Meaney, 2003):

(1) $$W=\frac{2\mu }{\alpha^{2}}\left(\lambda_{1}^{\alpha }+\lambda_{2}^{\alpha }+\lambda_{2}^{\alpha }-3\right)$$

where μ represents the shear modulus, α is a material-dependent parameter that introduces non-linear behavior, and λ_i_ are the principal stretches.

A satisfactory balance between simulation and tensile-testing based experimental data was achieved via an optimization process. The squared error between the simulated and experimental data was used as an objective function to be minimized. The stress-stretch curve obtained from an experiment is denoted as:

(2) $$S_{\exp}=S_{\exp}\left(\lambda \right)$$

where *S*_exp_ is the engineering stress and λ is the stretch of a sample from an uniaxial tensile test. Assuming the same experiment was simulated with FEM, the simulated stress-stretch curve would be denoted as:

(3) $$S_{\mathrm{sim}}=S_{\mathrm{sim}}\left(\lambda ,\alpha_{1},\ldots ,\alpha_{n}\right)$$

where *S*_sim_ is the simulated stress, λ is the prescribed stretch, and *a*_1_ through *a*_n_ are the material parameters to be determined. To optimize the material parameters, an error function, *y* = *S*_exp_ − *S*_sim_, is defined. The objective function to be minimized is *y*^2^ at pre-established values of the independent variable, λ, that fall within the test range. A practical approach is to discretize the stress-stretch curve and use a least-squared method to determine the parameters (Zhang and Gan, 2011). To demonstrate the application of the Pseudo-3D RVE, a single-variable example regarding a quasi-static uniaxial tensile-testing based experimental stress-stretch curve (Shreiber et al., 2009) combined with the FE simulation was created to determine the shear modulus, μ, of axons. It was assumed that both the axons and ECM follow the Ogden hyperelastic model in Eq. (1) (Meaney, 2003). The non-linear parameter, *α*, was set to be the nominal value, 8.22, obtained in Shreiber et al. (2009) via regression analysis of the experimental data. For simplicity, the same value is used for both the matrix and the axons, although it might be different for the axons and the matrix in general. The summation of the squared error of the stress-stretch curve at different pre-established values is used as the objective function. The problem can be described as:

(4) $$\begin{array}{ccc} & \text{Min}\;E\left(\mu \right)={\sum_{i=1}^{i=m}\left(S_{\exp\text{\_}i}-S_{\text{sim\_}i}\right)}^{2} & \\ & \text{s.t. }\mu_{\min}\leq \mu \leq \mu_{\max} \end{array}$$

where the squared error, *E*(μ), is the objective function, shear modulus, μ, is the material parameter to be optimized, *S*_exp_i_ and *S*_sim_i_ are experimental and simulated stress at stretch level λ^i^ the *i*^th^ pre-established stretch. Note that this is different from the principal stretch λ_i_.

**Figure 3:**
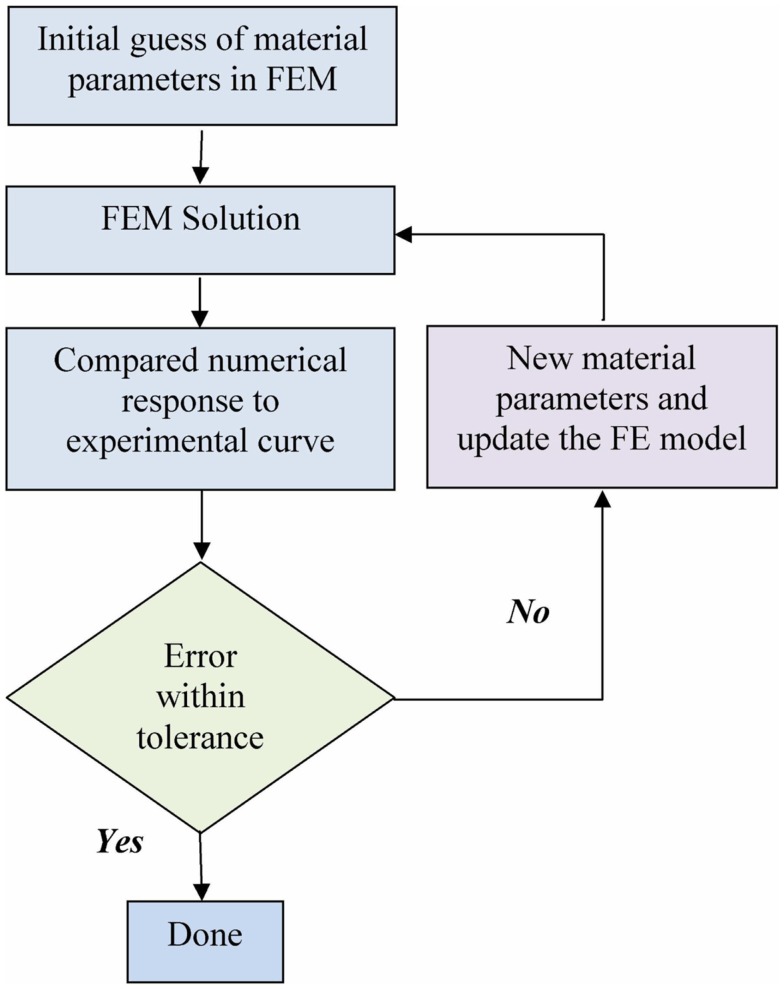
**Flow chart of the optimization process**.

The optimization for material properties is described in the flow chart in Figure 3. The optimization loop was achieved with a program coded in Python. The FE solver was used to solve the uniaxial tensile test numerically. The FE results were processed by the optimization program and compared to the experimental data. A new guess of parameters was generated based on the current comparison. The iteration is terminated when the optimized parameters were found using the tolerance of 1.0 kPa in the golden search algorithm.

## Results

**Figure 4:**
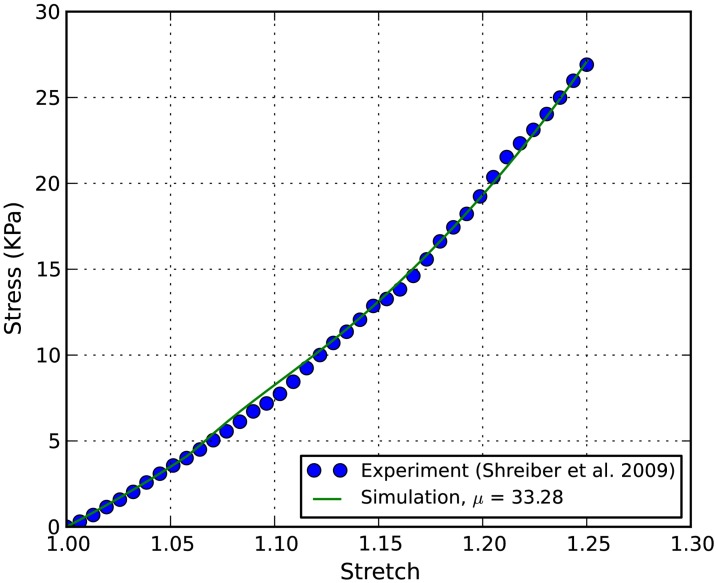
**Stress-stretch curves of E18 chick embryo spinal cord: the converged simulation vs. experimental in Shreiber et al. (2009)**.

The von Mises stress contours of the ECM and the axons subjected to a stretch rate of 1.06 in the *z*-axis are shown in Figures 2A,B, respectively. Since the interaction between matrixes and axons depends on the geometry of the axons, the stresses are highly localized. The overall average uniaxial stress-strain curves are plotted in Figure 4, where *S*_33_ is the nominal engineering stress component in the *z*-direction.

**Figure 5:**
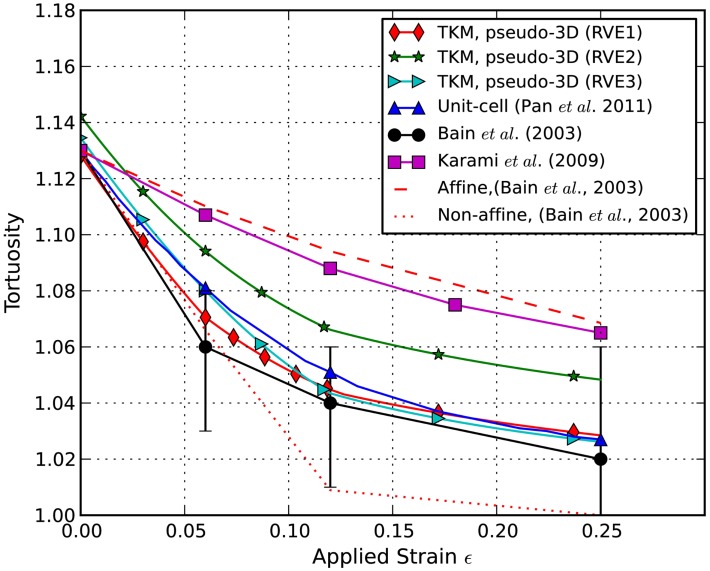
**Predicted evolution of undulation with stretch**. Simulation results are compared to experimental results from Bain et al. (2003) and to simulation results from Karami et al. (2009) and Pan et al. (2011).

The axons’ tortuosity is plotted against the applied stretch in Figure 5. It is shown that the undulated axons become less wavy when the applied stretch increases. In this pseudo-3D RVE model, the tortuosity decreased faster at smaller applied stretches as compared to other simulation models. The experimental data from Bain et al. (2003) and the simulation data from Karami et al. (2009), who employed a unit-cell method with fully constrained coupling between axons and glia, are included for comparison, as well as our results from our previous TKM using a unit-cell approach (Pan et al., 2011). Clearly, fractional coupling between the axons and glia yields more accurate stretch-undulation data than Karami et al.’s model with perfectly bonded axons and glia. The current pseudo-3D RVE approach also gives consistent axon kinematics. Curves obtained from RVE1 and RVE3 in this study are close to that from the unit-cell model and experimental measurements. The curve from RVE2 is slightly off the trend since its initial undulation was slightly higher than 1.13. In this case, the evolution of axonal coupling to matrix with stretch is delayed. A drawback of these simulations is that the percentage of axon embedment was held constant and only updated at discrete stretch levels, whereas in reality axon kinematics transition continuously. We expect that allowing the constraints to evolve with stretch will allow us to capture the non-affine, independent behavior of axons and glia at lower stretch levels and enable a more rapid change in undulation at low stretch levels.

In order to determine the material properties of white matter in tension, an inverse FE simulation using the RVE and experimental data was performed. The controlled experimental stress-stretch data of chick spinal cords in Shreiber et al. (2009) were extracted and plotted in Figure 4 for inverse FE simulation. The non-linear parameter α in the Ogden hyperelastic model was curve-fit to be 8.22 ± 1.27, which is used for both the axon and the ECM in the inverse simulation. The shear modulus of axon is assumed to be three times as stiff as that of ECM (Meaney, 2003).

**Figure 6:**
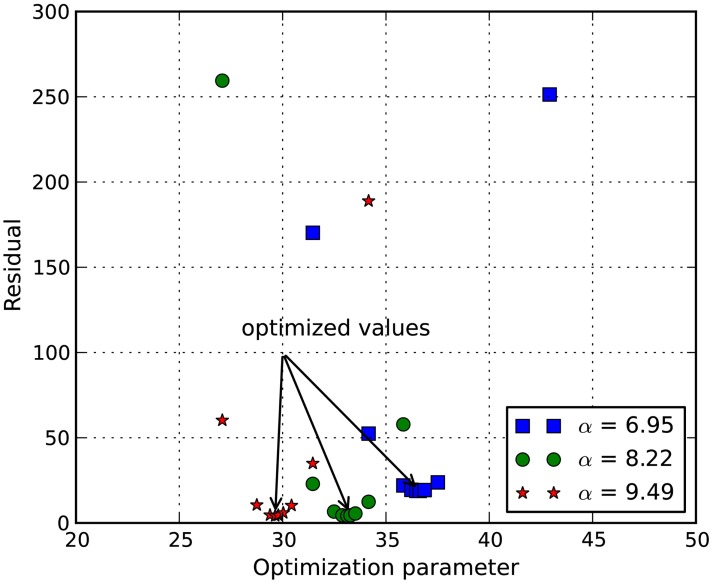
**Plot of function value at various shear modulus**. It shows that the squared error of the experimental curve and the simulated curve is minimized at μ = 36.63, 33.28, and 29.64 kPa for α = 6.95, 8.22, and 9.49, respectively.

**Figure 7:**
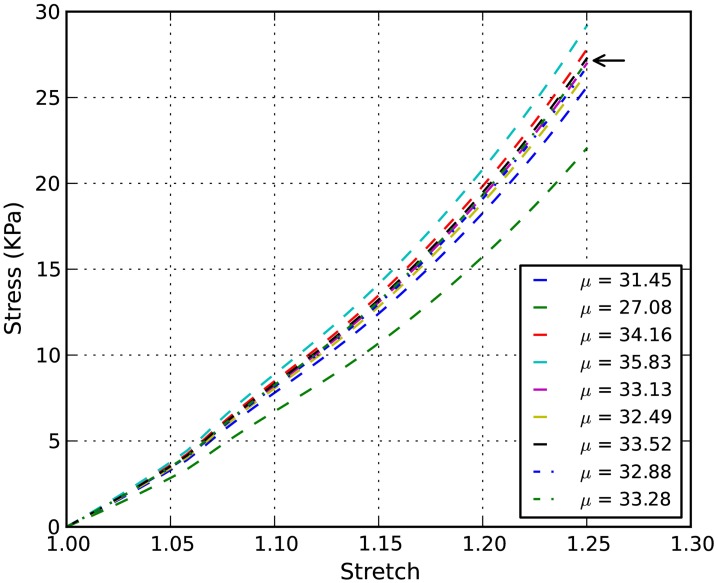
**Inverse simulation example: stress-stretch curves of chick embryo spinal cord with α = 8.22**. The arrow points to the stress-stretch curve corresponding to the optimized parameter μ = 33.28 kPa.

The stress-stretch curve was sampled at 20 equidistant intervals. The squared error was minimized with a golden search algorithm for this univariate problem in order to find the shear modulus of an axon, μ. An initial guess for μ was given for the algorithm. A pre-determined solution bracket (20, 50 kPa) was also provided to shorten the computational time. The solution tolerance was set to 1.0 kPa for the unknown parameter. The procedure was automated using Python scripts. After nine iterations, the optimized parameter μ = 33.28 kPa was obtained using the nominal value α = 8.22. The residual of each iteration was tabulated in Table 1 and plotted in Figure 6. It is shown that the unknown parameter converges to the optimized value as the residual reaches its minimum. The stress-stretch curves obtained in all iterations were plotted in Figure 7. The simulated stress-stretch curves converge to the experimental curve. To investigate the sensitivity of the results to the parameter α, the optimization process was also performed at α = 6.95 and 6.49. The respective optimized parameters are μ = 36.63 and 29.64 kPa and the respective residuals are tabulated in Table 1. The optimized parameters reside within the curve-fitting range of 32.8 ± 9.53 kPa obtained from the experimental data (Shreiber et al., 2009). It can be concluded that shear modulus is not sensitive to the non-linear parameter α since the change of μ is less than 11% and it is within the simulated range. The stress-stretch corresponding to the optimized parameter of μ = 33.28 kPa is also plotted in Figure 4 showing good agreement between the curves from the experiment and the optimization process.

**Table 1:** **Sensitivity analysis on the non-linear parameter α**.

Iteration	α = 6.95		α = 8.22		α = 9.49	
	μ	*E(μ)*	μ	*E(μ)*	μ	*E(μ)*
1	31.46	170. 20	31.46	22. 95	31.46	34. 92
2	42.92	251. 34	27.08	259. 45	27.08	60. 23
3	35.84	22. 00	34.16	12. 41	34.16	188. 86
4	34.16	52. 43	35.84	57. 83	29.79	4. 54
5	36.87	19. 3	33.13	4. 21	28.75	10. 55
6	37.51	23. 81	32.49	6. 69	30.43	10. 26
7	36.47	18. 83	33.52	5. 56	29.39	4. 62
8	36.23	19. 46	32.89	4. 48	30.03	5. 90
9	**36.63**	18. 79	**33.28**	4. 47	**29.64**	4. 20

*Change of the optimized parameter is within the range of ±11% when the parameter α varies within the experimental error range. *E*(μ) is the squared error between the experimental and the simulation values*.

*Bolded values are final results for μ with varying α*.

## Discussion

A novel pseudo-3D RVE with axon kinematics was developed to capture the kinematics of axons and the stress-stretch behavior of CNS white matter. The advantage of the embedded element model is that the complex geometry of the surrounding matrix is simplified since the volume of axons was not excluded from the matrix. The kinematics of axons can be retained using the simplified axon kinematic model, where a percentage of axons are transitioned from uncoupled to coupled based on the undulation and applied stretch. Stresses and strains are highly localized depending on the local undulation of axons and, importantly, on the interaction of the axon with the matrix. The effects of complex axon geometry on the local stress and strain fields are significant. The local stress and strain fields are highly heterogeneous nearby the axon, even though a uniform global strain field (ε_33_ = 0.06, 0.12, and 0.25 in this case) is applied to the boundary of RVE. This implies that if a mechanical strain on axons is used as an injury criterion, a macroscopically homogeneous model is not sensitive enough to capture the local variation and a local “sub-model” or “micro-model” has to be considered and implemented. With the help of high resolution MRI techniques (McNab et al., 2009), one may be able to obtain axon orientation of a human brain and build localized RVEs. The RVE will be useful to provide detailed, local axon deformation at the tissue-level. More work needs to be done to incorporate the model into a tissue-level representation of brain tissue white matter along this line.

The application of a pseudo-3D RVE was demonstrated in the inverse optimization of the shear modulus of a hyperelastic model for the chick embryo spinal cord. Limited data is available for the stress-strain properties of white matter in tension, and the chick embryo spinal cord studies clearly demonstrated that (1) axon kinematics transition from uncoupled to coupled with increasing stretch (Hao and Shreiber, 2007); and (2) coupling of axons to the glial matrix significantly affects the tensile stress-strain response (Shreiber et al., 2009). The E18 chick embryo spinal cords include gray and white matter, as well as the meningeal layers, and each may potentially contribute to the mechanical properties. If the spinal cord when tested in uniaxial tension is approximated as three concentric mechanical elements in a parallel composite, the dura, arachnoid, and pial maters are too thin in the chick embryo to provide any significant contribution to the mechanical response. At E18, the white matter comprises approximately 56% and the gray matter 44% of the cross-sectional area of the chick embryo spinal cord (Hao and Shreiber, 2007), so both tissues may contribute to the bulk tensile properties. However, Miller and Chiznei reported that brain tissue, with a predominance of gray matter with white matter that was not aligned with the direction of stretch, reaches a peak stress which is more than an order of magnitude lower than white matter in tension (Miller and Chinzei, 2002). Moreover, they found that stress-strain response of the brain tissue in tension was hypoelastic, contrary to the hyperelastic response of the chick embryo spinal cord (Shreiber et al., 2009) as well as the properties of the rat spinal cord (Fiford and Bilston, 2005). As such, the tensile properties of the spinal cords are expected to be dominated by the response of the white matter. Unsurprisingly, the simulated stress-stretch responses correlate well with the experimental results. The parameter obtained through the inverse FE optimization procedure agrees well with the fitted material parameter. It is noted that while only a univariate problem is shown in this paper, a non-linear least-squared method can be used for multiple-parameter problems.

The purpose of this study was to develop and validate a multi-scale computational approach that captures the microkinematic response of axons and the tissue-level response of the material in tension. The developing chick embryo spinal cord was chosen as our model tissue based on our previous characterizations. This chick embryo spinal cord conferred several advantages. Chick embryo tissue is readily available through procurement of fresh, fertilized eggs and is cost effective when compared to small mammals. Maintaining eggs during development is relatively simple, as is harvesting tissue at a desired developmental stage. Tissue can be rapidly harvested and tested – the experiments described in Shreiber et al. (2009) and Hao and Shreiber (2007) – were completed within an hour on tissue harvest, well within the window described in Gefen et al. (2003) and Gefen and Margulies (2004) for correlation of *ex vivo* to *in vivo* properties of CNS tissue. Similar to the optic nerve, which was tissue used for the original description of axon kinematics (Bain et al., 2003), axons in the spinal cord are predominantly oriented axially, the spinal cord also provides a system in which the prevailing direction of axons is parallel to the direction of stretch, which allows a direct interpretation of the relationship between macroscopic tissue stretch and microscopic axonal deformation. Along these lines, the fundamental behaviors observed in the chick embryo model paralleled those in the adult guinea pig optic nerve: undulated axons that straightened with stretch and demonstrated a transition from non-affine behavior at low stretch levels to affine behavior at high stretch levels. In addition, the form of the bulk stress-strain response of the chick tissue in tension matched that from rat spinal cord (Fiford and Bilston, 2005) and the adult guinea pig optic nerve (Bain et al., 1997; Bain and Meaney, 2000). As such, we believe that the model described herein is an accurate representation of CNS white matter in tension that can be applied to multi-scale analysis of TBI and SCI.

From the composite material mechanics of point of view, the mechanical properties of a composite heterogeneous material depend strongly on the volume fraction of the fiber; the higher the fiber volume fraction, the stiffer the composite material. In this study, the CNS white matter was treated as a biological fibrous composite material comprised of axons (fibers) and ECM (matrix). Changes of the average axon diameter will not affect the current results as long as the volume fraction remains unchanged. This is because the *X*- and *Y*-dimensions of the RVE need to be scaled proportionally to the axon diameter in order to maintain the same volume fraction. In this paper, we only consider a uniform axon diameter. It will be straightforward to adapt the methodology into the RVE with axon diameters that follow a distribution. In that case, a relative large number of axons are needed and the size of the RVE will increase.

To fully implement this multi-scale approach for analysis of human TBI or SCI, a number of parameters should be identified and/or tested. First, the tortuosity of axons in different regions of the brain and for different ages must be characterized. The chick embryo and guinea pig studies demonstrated that tortuosity changes with development and can differ between tissues/locations and/or species. Second, the RVE approach should be validated against other modes of mechanical loading. We focused on the multi-scale response of axons in white matter to tension because tensile strain has been recognized as the proximal cause of axonal damage (Margulies and Thibault, 1992). However, tension can arrive from any multi-axial, deviatoric loading condition, and it is important that the RVE approach accurately emulate the bulk response of tissue in shear and compression. Finally, the RVE should be used to simulate a model of injury to identify and compare to existing injury criteria; similarly to the guinea pig optic nerve characterization study (Bain et al., 2003) which was combined with *in vivo* injury studies (Bain and Meaney, 2000) to evaluate axon- and tissue-level injury criteria for functional damage, which was measured using electrophysiology, and morphological damage, which was identified with immunohistochemistry.

## Conflict of Interest Statement

The authors declare that the research was conducted in the absence of any commercial or financial relationships that could be construed as a potential conflict of interest.
